# Retraction: Determinants of Hypertension Among Patients With Type 2 Diabetes Mellitus in Karachi, Pakistan: A Cross-Sectional Study

**DOI:** 10.7759/cureus.r52

**Published:** 2022-03-17

**Authors:** Chamithra D Rupasinghe, Usama Shahbaz, Ellen Huang, Akshay Patel, Fares Mohammed Saeed Muthanna, Marina Basta, Chutimon Narawish, Sehrish Karim, Anum Rahim

**Affiliations:** 1 Internal Medicine, Manipal College of Medical Sciences, Pokhara, NPL; 2 Clinical Sciences, Saint James School of Medicine, The Quarter, AIA; 3 Internal Medicine, Oceania University of Medicine, Apia, WSM; 4 Internal Medicine, Saint James School of Medicine, The Quarter, AIA; 5 Pharmaceutical Care, Walailak University, Nakhon Si Thammarat, THA; 6 School of Medicine, St. George's University, True Blue, GRD; 7 College of Medicine, Rangsit University, Pathum Thani, THA; 8 Public Health Sciences, Aga Khan University Hospital, Karachi, PAK; 9 Indus Hospital Research Center, The Indus Hospital, Karachi, PAK

This article has been retracted due to the unknown origin of the data, lack of verified IRB approval, and purchased authorships. While not listed as an author, it was discovered that Rahil Barkat wrote and coordinated the submission of this article. Mr. Barkat was involved in data theft and misuse in two recently published Cureus articles, which have since been retracted.

As the origin of this article’s data and verified IRB approval cannot be confirmed, we have made the decision to retract this article. Cureus has confirmed that the co-authors were asked by Mr. Barkat to proofread the article and provide payment in exchange for authorship. (Proofreading is an insufficient contribution to warrant authorship as defined by ICMJE.) These payments were made in the guise of “editing fees” but greatly exceed any editing fees paid to Cureus. While these authors may have been defrauded by Mr. Barkat, they remain complicit due to their lack of honest contributions to the article.

